# Correlates of Complete Childhood Vaccination in East African Countries

**DOI:** 10.1371/journal.pone.0095709

**Published:** 2014-04-21

**Authors:** Maureen E. Canavan, Heather L. Sipsma, Getnet M. Kassie, Elizabeth H. Bradley

**Affiliations:** 1 Yale School of Public Health, New Haven, Connecticut, United States of America; 2 University of Addis Ababa, Addis Ababa, Ethiopia; Melbourne School of Population Health, Australia

## Abstract

**Background:**

Despite the benefits of childhood vaccinations, vaccination rates in low-income countries (LICs) vary widely. Increasing coverage of vaccines to 90% in the poorest countries over the next 10 years has been estimated to prevent 426 million cases of illness and avert nearly 6.4 million childhood deaths worldwide. Consequently, we sought to provide a comprehensive examination of contemporary vaccination patterns in East Africa and to identify common and country-specific barriers to complete childhood vaccination.

**Methods:**

Using data from the Demographic and Health Surveys (DHS) for Burundi, Ethiopia, Kenya, Rwanda, Tanzania, and Uganda, we looked at the prevalence of complete vaccination for polio, measles, Bacillus Calmette–Guérin (BCG) and DTwPHibHep (DTP) as recommended by the WHO among children ages 12 to 23 months. We conducted multivariable logistic regression within each country to estimate associations between complete vaccination status and health care access and sociodemographic variables using backwards stepwise regression.

**Results:**

Vaccination varied significantly by country. In all countries, the majority of children received at least one dose of a WHO recommended vaccine; however, in Ethiopia, Tanzania, and Uganda less than 50% of children received a complete schedule of recommended vaccines. Being delivered in a public or private institution compared with being delivered at home was associated with increased odds of complete vaccination status. Sociodemographic covariates were not consistently associated with complete vaccination status across countries.

**Conclusions:**

Although no consistent set of predictors accounted for complete vaccination status, we observed differences based on region and the location of delivery. These differences point to the need to examine the historical, political, and economic context of each country in order to maximize vaccination coverage. Vaccination against these childhood diseases is a critical step towards reaching the Millennium Development Goal of reducing under-five mortality by two-thirds by 2015 and thus should be a global priority.

## Background

Despite the health and economic benefits of childhood vaccinations [1]–[5], vaccination rates in low-income countries (LICs) vary widely. Less than half of children globally receive complete vaccination coverage [6], and this may be an overestimate as officially reported vaccination rates can mask irregularities in how vaccinations are delivered, limiting their effective coverage [7]. Inadequate coverage leaves children vulnerable to unnecessarily high rates of morbidity and mortality [8], [9]. Increasing coverage of vaccines in the poorest countries to 90% in the next 10 years has been estimated to prevent 426 million cases of illness and avert nearly 6.4 million childhood deaths worldwide [2], [3].

Current literature has identified several barriers to vaccinations in LICs, including lower parental education [10]–[12], lower maternal age [10], lower income [4], [11]–[14] female gender of the child[14]–[16], traditional or Muslim religion [17], [18], and larger family size [4], [15]. Most existing studies, however, have used small or non-representative selected samples, were conducted nearly a decade ago, or have examined only a subset of all recommended vaccinations. Only three studies use recent nationally representative data from the household-based Demographic and Health Surveys (DHS), and each has substantial shortcomings. Two of these studies, for example, focus on only a single country [19], [20]. The other study focuses on receiving the first, rather than the complete, dose of DTP (diphtheria-pertussis tetanus vaccine)vaccine as recommended by the WHO and does not provide an estimation of complete vaccination coverage [21].

Consequently, we sought to provide a comprehensive examination of complete vaccination status in East Africa. We use nationally representative data from the DHS for six East African countries to identify common and country-specific barriers to complete childhood vaccination status as recommended by the WHO vaccination schedule. These results can be used as evidence to design interventions for achieving the Millennium Development Goal of at least 90% vaccination within these countries [22].

## Methods

### Ethics Statement

This study used publicly available data from the Demographic Health Surveys and thus was exempt from review by the Human Investigation Committee (HIC) because no identifying participant information was obtained. Prior to receiving access to the data, an electronic registration form highlighting the desired data and plan for analysis was submitted and approved by the DHS. All data were reported in aggregate and no attempts were made to identify any study participants.

### Study Design and Sample

We conducted a cross-sectional analysis using self-report data from the most recent versions of the Demographic and Health Surveys (DHS) for the following East African countries: Burundi (2010), Ethiopia (2011), Kenya (2009), Rwanda (2010), Tanzania (2010) and Uganda (2011). We used this sample to ensure comparability across countries; their data were all collected within the past 3 years using the standard version of the DHS.

These nationally representative surveys were designed to measure population health and nutrition information. All DHS surveys employed a two-stage cluster sample design. Enumeration areas (EA) were first randomly selected from census files stratified by region and urban/rural status, and then a sample of households were randomly selected within each EA. DHS data for each country include a women’s questionnaire that measures sociodemographic characteristics of the mother, information on reproductive health and behavior, as well as child-specific information for all births within the past five years for women of reproductive age (15–49 years).

In order to measure complete vaccination for children up to 12 months of age, we followed a widely used strategy [6], [7], [19], [23]. We first limited our sample to all children between 12 and 23 months (784 in Burundi; 1,889 in Ethiopia; 1,091 in Kenya; 794 in Rwanda; 1,526 in Tanzania; and 481 in in Uganda). We then restricted the sample to children with valid responses on our outcome measure and our independent variables, excluding 7 in Burundi, 70 in Ethiopia, 55 in Kenya, 3 in Rwanda, 15 in Tanzania, and 7 in in Uganda. Our final analytic samples included the following numbers of individuals from each country (response rate calculated out of the total population of living children ages 12–23 months): 777 (99.1%) in Burundi; 1,819 (96.3%) in Ethiopia; 1,036 (95.0%) in Kenya; 791 (99.0%) in Rwanda; 1,511 (99.0%) in Tanzania; and 474 (98.5%) in Uganda.

### Measures

#### Outcome

Our primary outcome measure was complete vaccination as recommended by the WHO vaccination schedule [24]. Complete vaccination was described as having received all doses for each of 4 vaccines: polio (4 doses administered at Birth, 6, 10, and 14 weeks), measles (administered at 9 months), Bacillus Calmette–Guérin (BCG) against tuberculosis (administered at Birth), and DTwPHibHep (DTP) against diphtheria, tetanus, pertussis, influenza, and hepatitis B (3 doses administered at 6, 10, and 14 weeks). This variable was created using several items for each child. Respondents were first asked if they had a health card (with dates and types of vaccines administered) for each child. If they were not able to produce a health card or if the health card was missing vaccination information, respondents were asked if their child received each of these vaccines and the number of times each of these vaccines were given. We classified vaccination status based on responses to these questions into the following 4 distinct categories: received complete vaccinations with proof of date of vaccination by 12 months (confirmed using the health card); received complete vaccinations without proof of date (the health card did not provide a date or there was no record of the vaccination but the mother responded that the child received the vaccine); received incomplete vaccinations (any but not all recommended doses, regardless of whether or not a health card was present or a date of vaccination could be determined); or did not receive any of the vaccinations.

#### Independent variables

We incorporated several variables about health care access and sociodemographic characteristics based on hypothesized and previously demonstrated associations in the literature[4], [10]–[12], [14], [17], [25], [26]. Our analysis used two variables to specifically measure health care access: location of delivery (home; public institution, hospital or health center; private institution, hospital or health center; other type of institution) and whether or not the child received a check-up within 2 months of birth. Our sociodemographic variables included child’s sex, the mother’s marital status (married, never married, formerly married), education (none, primary or less, secondary and above) age, and the mother’s current employment status (employed versus not employed). We also included family size (the number of living children in the household), religion (Christian, Muslim or other), household poverty status (being in the poorest wealth quintile or not), location of residence (urban versus rural), and the region of the country where the mother lived.

### Data Analysis

We generated frequencies to describe the distribution covariates and complete vaccination status by region for all countries. Unadjusted associations between complete vaccination status and all independent variables were estimated using chi-square tests. We conducted multivariable logistic regression within each country to estimate associations between complete vaccination status according to the WHO schedule (with proof of date) and independent variables.

Prior to fitting the multivariable model, we assessed potential multi-collinearity among independent variables using tolerance and variance inflation factor cut points of 0.1 and 4.0 respectively, as recommended [27]. To estimate the multivariable model, we included all variables that were significant (P-value <0.10) in our unadjusted analysis or that were hypothesized to be associated with vaccination rates. We then one by one eliminated variables that were non-significant (P-value >0.05) in the multivariable model, beginning with the highest P-value using previously described methods [28], [29]. At each iteration we assessed model fit [29] and confirmed that variable removal did not change any remaining parameter estimates by more than 20% to account for potential confounding following the strategy used by Maldonado and colleagues [30]. We accounted for possible correlations among children within a single household by adjusting for clustering at the household level in all analyses. Additionally, all analyses were weighted and accounted for the complex survey design. We used SAS software, version 9.2 (SAS institute, Cary, NC) to perform all analyses.

## Results

### Sample Characteristics

**Figure 1:**
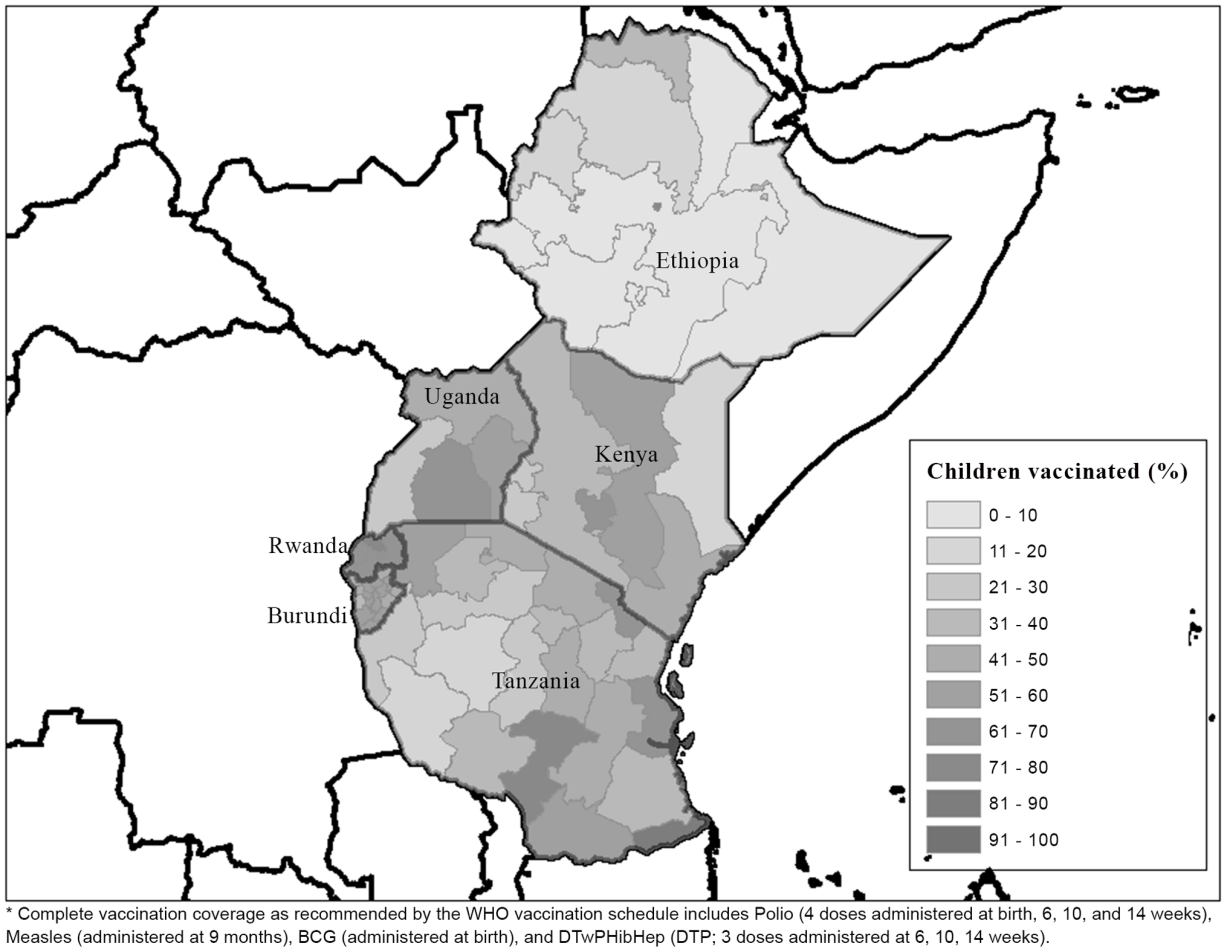
Regional variation in the proportion of children ages 12-23 months receiving complete vaccination*.

Vaccination status distributions varied significantly by country (P-value <0.01; **Table 1**). Rwanda had the highest proportion of children who received the complete WHO recommended vaccination schedule (66.2%); Ethiopia the lowest proportion of children with complete vaccination status (13.3%). In all countries, the majority of children received at least one dose of a WHO recommended vaccine; however, in Ethiopia, Tanzania, and Uganda less than 50% of children received a complete schedule of recommended vaccines. Within countries, regional variation was also apparent (**Figure 1**). Significant regional variation in children with complete vaccination status was present in Ethiopia, Kenya, Tanzania, and Uganda.

**Table 1:** Distribution of vaccination coverage as recommended by the WHO vaccination schedule* by country among children ages 12–23 months.

	Burundi	Ethiopia	Kenya	Rwanda	Tanzania	Uganda	
	N = 777^1^	N = 1,819^1^	N = 1,036^1^	N = 791^1^	N = 1,511^1^	N = 474^1^	
	%	%	%	%	%	%	P-value^^2^^
**Recommended vaccinations received**							<0.001
** All with proof of date**	47.2	13.3	38.6	66.2	38.8	27.6	
** All without proof of date**	29.4	11.6	14.1	14.4	7.3	10.1	
** Incomplete**	22.5	59.8	44.0	19.3	51.6	58.4	
** None**	1.0	15.3	3.3	0.1	2.3	3.8	

*Complete vaccination coverage as recommended by the WHO vaccination schedule includes Polio (4 doses administered at Birth, 6, 10, 14 weeks), Measles (administered at 9 months), BCG (administered at birth), and DTwPHibHep (DTP) (3 doses administered at 6, 10, 14 weeks).

1 Unweighted values.

^2^P-values correspond to χ^2^ tests used to determine statistically significant differences by country in the percentages of children ages 12–23 months who received the complete WHO recommended vaccination schedule.

### Factors Associated with Complete Vaccination Status

In unadjusted analysis, the likelihood of complete vaccination status was associated with place of delivery in all countries except in Burundi (**Table 2**), with complete vaccination status being more likely among those who were delivered in a public facility versus at home. Increased likelihood of complete vaccination status was also associated with getting a check-up within 2 months of birth in Burundi, Kenya, and Uganda, although not in the other countries. Sociodemographic factors including maternal employment, marital status (currently married, never married, or formerly married), smaller family size, and higher education were associated with increased likelihood of complete vaccination status for children in Burundi, Ethiopia, Kenya, Tanzania, and Uganda. We found no significant differences in receiving complete vaccination by child’s sex, mother’s age, or religion in any country.

**Table 2:** Distribution of sample characteristics among children ages 12–23 months.

	Burundi	Ethiopia	Kenya	Rwanda	Tanzania	Uganda
	N = 777	N = 1,819	N = 1,036	N = 791	N = 1,511	N = 474
	%	%	%	%	%	%
Urban residence						
Yes	9.0	14.0	22.6	10.0	19.5	12.2
No	91.0	86.0	77.4	90.0	80.5	87.8
Poor wealth status						
Yes	42.1	44.5	42.7	44.9	45.0	44.0
No	57.9	55.5	57.3	55.1	55.0	56.0
Mother’s marital status						
Never married	2.4	0.6	8.8	8.1	5.9	3.8
Currently married	91.9	92.9	85.3	82.4	85.0	86.8
Formerly married	5.7	6.5	5.9	9.5	9.2	9.4
Mother’s education						
No formal education	54.0	68.5	11.3	16.3	24.7	12.3
Primary school	39.5	26.5	64.4	76.0	68.3	65.8
Secondary school or higher	6.5	5.0	24.3	7.7	7.0	22.0
Mother currently working						
Yes	89.1	53.7	57.6	90.1	90.9	77.1
No	10.9	46.3	42.4	9.9	9.1	22.9
Family Size*	3.34 (0.08)	3.45 (0.05)	3.28 (0.08)	3.0 (0.07)	3.6 (0.06)	3.75 (0.12)
Child received check-upwithin 2 months of birth						
Yes	11.4	3.6	36.6	5.3	11.7	29.5
No	88.6	96.4	63.4	94.7	88.3	70.5
Location of delivery						
Home	32.0	87.5	53.9	20.8	48.6	41.3
Public institution	59.3	10.6	35.3	76.3	40.7	45.1
Private institution	4.8	1.2	10.7	0.6	7.0	12.3
Other institution	4.0	0.7	0.1	2.3	3.6	1.3

*Mean (SD) are presented for continuous variables.

Consistent with the unadjusted analysis, in multivariable analysis, we found that being delivered in a public institution compared with being delivered at home was associated with increased odds of complete vaccination status in all countries except Burundi. In Ethiopia and Kenya, effect sizes were quite large, with children who had been delivered in a public institution versus at home having more than twice the odds of complete vaccination status according to the WHO schedule (**Table 3**). In Rwanda, children delivered in private and public institutions versus at home were 1.75 and 9.05 times as likely to have complete vaccination status, respectively. Similarly, children delivered in a private or public institution were more likely to have complete vaccination status compared with children delivered at home in Tanzania (adjusted OR for private versus home delivery = 2.77, CI: 2.01, 3.83; adjusted OR for public versus home delivery = 2.11, CI: 1.09, 4.08, respectively ) and in Uganda (adjusted OR for private versus home delivery = 4.17, CI: 2.34, 7.42; adjusted OR for public versus home delivery = 3.46, CI: 1.48, 8.08, respectively).

**Table 3:** Correlates of complete vaccination status as recommended by the WHO vaccination schedule among children ages 12–23 months¥.

	Burundi	Ethiopia	Kenya	Rwanda	Tanzania	Uganda
	N = 777	N = 1,819	N = 1,036	N = 791	N = 1,511	N = 474
Urban residence	–	2.23 (1.13, 4.43)*	–	–	2.60 (1.66, 4.07)*	–
Poor wealth status	–	0.54 (0.33, 0.86)*	–	–	–	–
Mother’s marital status						
Currently married	–	–	Reference	–	Reference	–
Never married	–	–	0.87 (0.41, 1.83)	–	0.75 (0.41, 1.36)	–
Formerly married	–	–	0.43 (0.20, 0.92)*	–	0.46 (0.27, 0.78)*	–
Mother’s education						–
No formal education	–	–	–	–	–	Reference
Primary school	–	–	–	–	–	1.49 (0.63, 3.54)
Secondary school or higher	–	–	–	–	–	3.39 (1.20, 9.51)*
Mother currently working	1.98 (1.21, 3.23)*	–	–	–	–	–
Family Size	–	–	0.88 (0.79, 0.99)*	–	–	0.90 (0.81, 1.00)*
Child received check-upwithin 2 months of birth	0.52 (0.32, 0.85)*	–	–	–	1.95 (1.21, 3.16)*	–
Location of delivery						
Home	–	Reference	Reference	Reference	Reference	Reference
Public institution	–	3.11 (1.62, 5.98)*	2.41 (1.64, 3.56)*	1.75 (1.21, 2.54)*	2.77 (2.01, 3.83)*	3.94 (2.12, 7.33)*
Private institution	–	1.92 (0.66, 5.56)	1.40 (0.78, 2.50)	9.02 (1.06, 76.87)*	2.11 (1.09, 4.06)*	3.13 (1.34, 7.33)*
Other institution	–	0.39 (0.05, 3.07)	–	0.54 (0.20, 1.46)	2.13 (1.03, 4.41)*	2.37 (0.31, 18.13)

¥ Model identifies variables significantly associated with complete vaccination after performing backwards stepwise elimination with a cut-point of 0.05 for model retention. Ethiopia, Kenya, Tanzania and Uganda are also adjusted for significant regional variation within each country.

*P-value <0.05.

Whether or not the child received a check-up within 2 months of birth was also associated with complete vaccination status in two countries. Children who received a check-up within 2 month of birth had nearly half the odds of complete vaccination according to the WHO vaccination schedule in Burundi (adjusted OR = 0.52, CI: 0.32, 0.83), but in Tanzania, children receiving a check-up within 2 months of birth had significantly greater odds of complete vaccination (adjusted OR = 1.95, CI: 1.21, 3.15). Additional sociodemographic covariates including maternal education, maternal marital status, maternal employment, family size, urban residence, and poor household wealth were not consistently associated with complete vaccination status across countries.

We also conducted multinomial logistic regression to examine vaccination with proof of date and without proof of date compared with incomplete/no vaccination; results did not differ significantly from those presented for complete vaccination with proof of date. Additionally, we found no significant differences between children with and without proof of date. We therefore decided to use the more conservative estimate for complete vaccination status according to the WHO recommended schedule.

## Discussion

Overall, complete vaccination status rates according to the WHO vaccination schedule were low (mean = 39%), highlighting the need for improved efforts to ensure complete and consistent vaccination in each country. Complete vaccination status, however, varied significantly among the East African countries in our sample, ranging from 13% in Ethiopia to 66% in Rwanda, and thus suggests a wide range in the quality and effectiveness of the healthcare infrastructure in East Africa. However, the majority of children received at least some of the recommended vaccines, suggesting some exposure to the health care system.

Although multivariable regression showed no consistent set of predictors accounting for complete vaccination status, we observed differences in vaccination coverage based on region and the location of delivery. These differences point to the need to examine the historical, political, and economic context of each country in order to maximize vaccination coverage. Additionally, we did not find a significant effect for poverty on complete vaccination status for every country included in our sample. The poverty effect may be have been explained by other covariates, particularly those related to health care access, such as region. Regional variation could indicate differential access to vaccinations due to either transportation issues [31], [32] or difficulties in supply chain coverage or presence of demand related barriers [33]. Factors including staffing shortages, management of health facilities, quality of care available, and community communication can also account for differences in quality of service delivery and therefore may account for differences in vaccination coverage [34]–[36]. Country-specific examples have found that health facility factors, such as waiting times at facilities, are associated with rates of vaccination [10]. Improving vaccination access across all regions of a country could have a multifaceted effect on healthcare delivery by increasing health worker productivity and coverage area as well as the maximizing the efficiency of their health interventions [31].

The health sector should provide mothers with information regarding access to vaccinations and education on their importance prior to delivery. Because children delivered in public and private institutions were more likely to have complete vaccinations in several countries, initial contact with the health care system may be a crucial point for instilling the importance of vaccination coverage and setting up a referral or monitoring system for children and post-natal care [37]. Additionally, it highlights the importance of tailoring interventions to improve vaccination coverage so that they reach the populations in need. Our results further suggest that the current infrastructure may not do well to capture children delivered at home; as long as women continue to deliver at home, special attention should be given to reach these children and provide them with the necessary vaccinations.

Despite the advantages of contact with the health care system for both antenatal and postnatal care, including vaccinations, many women in low-income countries do not have steady contact with the health care system for multiple reasons. Results from our analysis showed that children in Burundi were significantly less likely to have complete vaccination coverage according to the WHO schedule if they received a check-up within 2 months after birth. Although this seems counterintuitive, research suggests that perception of the quality of health services in Burundi is quite low [38]. In addition to issues within the health care system including understaffing [39], [40], stock shortages of essential medications, and poor health information systems [37], [39], a large proportion of children in Burundi suffer from malnutrition and high poverty [39]. Recent qualitative data from Ethiopia confirms that concerns about the health care industry in terms of inadequate equipment and staffing at health centers is often a barrier for women to seek antenatal or postnatal care [41], [42]. Additionally, Burundi might have a more effective process for supply chain delivery or education for home based births and prenatal care that lead to higher vaccination rates for children among mothers who deliver at home. Recent studies have demonstrated that the use of lay health workers, compared with usual care, was associated with increased childhood vaccination rates [43], [44].

Given the benefits of vaccination in children in terms of morbidity [45], [46] and mortality [9], governments have incentive to increase vaccination rates. Studies have documented that government estimates provide an inflated estimation of vaccine coverage due to relying on non-representative samples and counting incomplete vaccines in their estimates [7]. Consequently, many countries may not understand the factors that are associated with complete vaccination according to the WHO schedule within their countries. In our analysis, we found that across the East African countries many children received incomplete vaccination according to the WHO schedule. Our results build upon the need to use a standard set of guidelines and data source when reporting of vaccination coverage. Using a standard instrument and a consistent definition of complete coverage allows us to accurately estimate the true burden of unmet need for complete vaccination and to compare vaccination rates across countries. The DHS is an ideal data source because it uses standardized instruments and provides detailed descriptions of the changes made between versions with recodes to ensure continuity. Additionally, though not the gold standard, data obtained via the DHS is nationally representative capturing vaccines delivered by both private and public agencies. Recent studies suggest substantial overreporting of individual vaccines coverage across low- and middle-income countries (LMIC) due to inconsistent data sources [6], [47]. The implications of overreporting vaccination rates could correspond to a reduction in over 100 million children not vaccinated against the WHO recommended diseases across all LMICs [6].

Our findings should be interpreted in light of several limitations. First, we used a conservative estimate that complete vaccination status had to have a health card with time documentation. Although this allows for the potential of underestimating vaccination coverage by not including vaccinations that had missing health cards, it provided an objective measurement and we found no significant difference in the distribution of independent variables among the complete vaccination groups. There is also the potential for bias due to missing responses for vaccination status; however, this type of bias is not likely to be substantial due to the high response rates for complete vaccination status. Additionally, we used cross-sectional data and are only able to make conclusions about associations with complete vaccinations; causality cannot be ascertained from our data. Moreover, we were unable to adequately investigate regional and district differences in rates of complete vaccination status because DHS data are sampled to be representative at a national and regional level only and is not conducive to smaller area estimation. Future studies using data collected for a more discrete analysis level are needed to delve into these differences and to highlight potential barriers to vaccination at more local levels. Finally, our analysis is limited in its ability to provide sufficient explanation for variations in complete vaccination across sample countries and regions due to inadequate data on the contextual differences between these East African countries; further studies are warranted to identify the specific difference in these relationships as well as what patterns may exist for other parts of Africa.

## Conclusion

In spite of the health and economic benefits of complete childhood vaccinations, we found complete vaccination status according to the WHO vaccination schedule was well below the 90% target for reducing the burden of childhood illness and death and varied considerably by country. Vaccination against these childhood diseases is a critical step towards reaching the Millennium Development Goal of reducing under-five mortality by two thirds by 2015 and thus should be a global priority. This gap highlights the need for improved consistent access to the healthcare system in each country which can only be achieved by examining the historical, political, and economic landscape of each country and how to address the specific barriers to health care use within each country in order to maximize vaccination coverage.
